# Protein Modification by Strain-Promoted Alkyne–Nitrone Cycloaddition**

**DOI:** 10.1002/anie.201000408

**Published:** 2010-03-23

**Authors:** Xinghai Ning, Rinske P Temming, Jan Dommerholt, Jun Guo, Daniel B Ania, Marjoke F Debets, Margreet A Wolfert, Geert-Jan Boons, Floris L van Delft

**Affiliations:** Complex Carbohydrate Research Center, University of Georgia315 Riverbend Road, Athens, GA 30602 (USA); Radboud University Nijmegen, Institute for Molecules and MaterialsHeijendaalseweg 135, 6525 AJ, Nijmegen (The Netherlands)

**Keywords:** cycloaddition, cycloalkynes, kinetics, nitrones, protein modifications

The bioorthogonal chemical reporter strategy is emerging as a versatile method for the labeling of biomolecules, such as nucleic acids, lipids, carbohydrates, and proteins.^1^ In this approach, an abiotic chemical functionality (reporter) is incorporated into a target biomolecule and can then react with a complementary bioorthogonal functional group linked to one of a diverse set of probes.

The azide functional group, which is the most commonly employed reporter, can react in a Staudinger ligation with modified phosphines,^2^ in a copper(I)-catalyzed cycloaddition with terminal alkynes (CuAAC),^3^ or in a strain-promoted alkyne–azide cycloaddition (SPAAC).^4^ The last type of reaction^5^ is attractive because it does not require a cytotoxic metal catalyst and therefore provides unique opportunities for the labeling of cell-surface glycans4b, 6 and proteins^7^ of living cells, the decoration of polymeric nanostructures,^8^ the labeling of lipids,^9^ proteomics,^10^ and tissue reengineering.^11^

**Figure 1:**
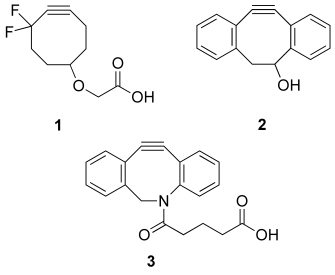
Ring-strained cyclooctynes for bioorthogonal cycloaddition reactions with azides.

The first generation of cyclooctynes suffered from relatively slow reaction rates; however, it has been found that the rate of strain-promoted cycloaddition can be increased by appending electron-withdrawing groups adjacent to the triple bond. For example, reactions of difluorinated cyclooctynes, such as **1** (Figure 1), with azides proceed approximately 60 times faster than the corresponding reactions of unsubstituted derivatives.5a We have found that derivatives of the 4-dibenzocyclooctynol **2** react fast with azido-containing saccharides and amino acids and can be employed for the visualization of metabolically labeled glycans of living cells.^12^ Attractive features of dibenzocyclooctynols include easy synthetic access, nontoxicity, and the straightforward attachment of a variety of probes. Recently, we introduced the more polar azacyclooctyne **3**,^13^ which exhibits a higher rate of reaction. Despite these advances, there is an urgent need for new and faster bioorthogonal reactions for labeling at low concentration.1b

We report herein a novel bioorthogonal reaction pair based on strain-promoted alkyne–nitrone cycloaddition (SPANC) to give N*-*alkylated isoxazolines with exceptionally fast reaction kinetics. The new methodology was used in a one-pot three-step protocol for the site-specific modification of peptides and proteins.

Nitrones **4 a**–**f** were readily prepared by the condensation of appropriate aldehydes with *N*-methylhydroxylamine. Cycloaddition reactions of **4 a**–**f** with cyclooctynol **2** in a mixture of acetonitrile and water gave the corresponding stable^14^ isoxazolines, in most cases in high yield (Table 1). We measured the rate constants of the cycloaddition reactions by ^1^H NMR or UV spectroscopy at 25 °C and found that the substituents on the nitrone greatly influenced the reaction kinetics. For example, the replacement of an *N-*methyl with a phenyl group (to give **4 c**) led to a faster reaction,^15^ whereas nitrone **4 d**, derived from a ketone, exhibited reaction kinetics that were too slow for accurate determination of the rate constant. Exceptionally high reaction rates were measured for the cycloaddition of **2** with α-carboxynitrones **4 e** and **4 f**. These reactions proceeded 18 and 32 times as fast, respectively, as the cycloaddition of **2** with benzyl azide (0.12 m^−1^ s^−1^).^16^ Also, we found that a high water content increased the reaction rate constants (e.g. 12.8 m^−1^ s^−1^ for a derivative of **2** in acetonitrile/water (1:9); see the Supporting Information).^17^ Finally, we determined a rate constant for the cycloaddition of azacyclooctyne **3** with **4 f**. As expected,^13^ a further enhancement of the reaction rate (39 m^−1^ s^−1^) was observed when **3** was used in place of the carbon analogue **2**.

**Table 1:** Rate constants for the cycloaddition of dibenzocyclooctynol **2** with nitrones **4 a**–**f**.[a]

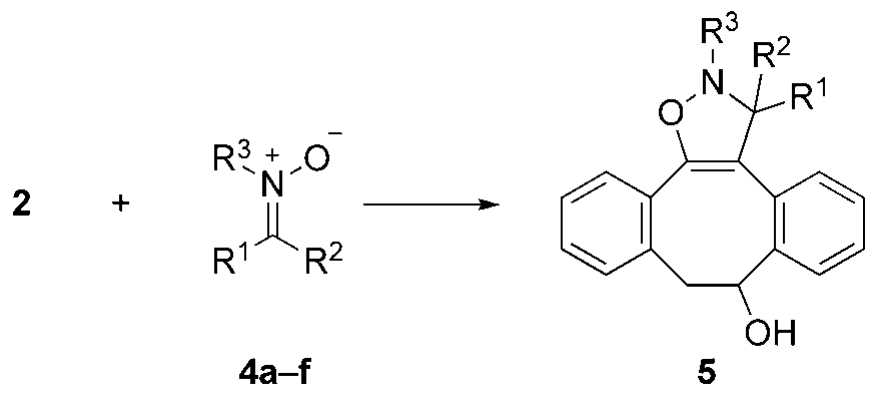						
**4**	R^1^	R^2^	R^3^	*k*[b] [m^−1^ s^−1^]	*k*′	Yield [%]
**a**	H	Ph	Me	1.3×10^−2^	1	95
**b**	H	CH_2_CH_2_Ph	Me	3.2×10^−2^	3	80
**c**	H	Ph	Ph	>0.2[c]	>17	89
**d**	Me	CH_2_CH_2_CO_2_Et	Me	<1×10^−3^	<0.1	33
**e**	H	CO_2_Et	Me	3.9	330	92
**f**	H	C(O)NHBn	Me	2.2	180	93

[a] The nitrone substrates (except **4 d**) were formed as pure *Z* isomers. Isoxazoline **5** was formed as a mixture of regio- and diastereoisomers. See the Supporting Information for reaction conditions.

[b] Method A: The rate constant was determined by ^1^H NMR spectroscopy in CD_3_CN/D_2_O (3:1); [**2 a**]=18 mm, [**4 a**–**f**]=16.4 mm. Method B: The rate constant was determined^18^ by UV spectroscopy in CH_3_CN/H_2_O (3:1); [**2 a**]=0.33 mm, [**4 a**–**f**]=0.30 mm. These reactions were too fast for monitoring by NMR spectroscopy.

[c] The reaction was too fast for accurate determination of the rate constant by NMR spectroscopy. Determination by UV spectroscopy was not possible owing to overlapping absorptions. Bn=benzyl.

**Figure 2:**
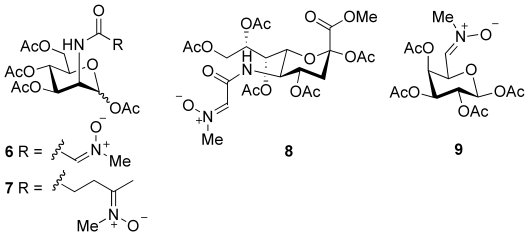
Nitrone derivatives of D-mannosamine (compounds **6** and **7**), sialic acid (compound **8**), and D-galactose (compound **9**) for metabolic cell-surface labeling.

Next, the challenge was to find a strategy for the incorporation of nitrones into biomolecules. We first focused our attention on metabolic labeling with monosaccharide derivatives bearing a nitrone moiety.^19^ Unfortunately, the incubation of Jurkat cells in the presence of nitrones **6**–**9** (10, 20, 50, and 100 μm; Figure 2), followed by labeling with dibenzocyclooctyne–biotin and staining with an avidin–fluorescein isothiocyanate (FITC) conjugate, led to no detectable fluorescence labeling of the cells.^12^ Presumably, either the biosynthetic glycosylation machinery does not accept nitrone modifications, or nitrones undergo intracellular hydrolysis in acidic compartments.

**Scheme 1:**
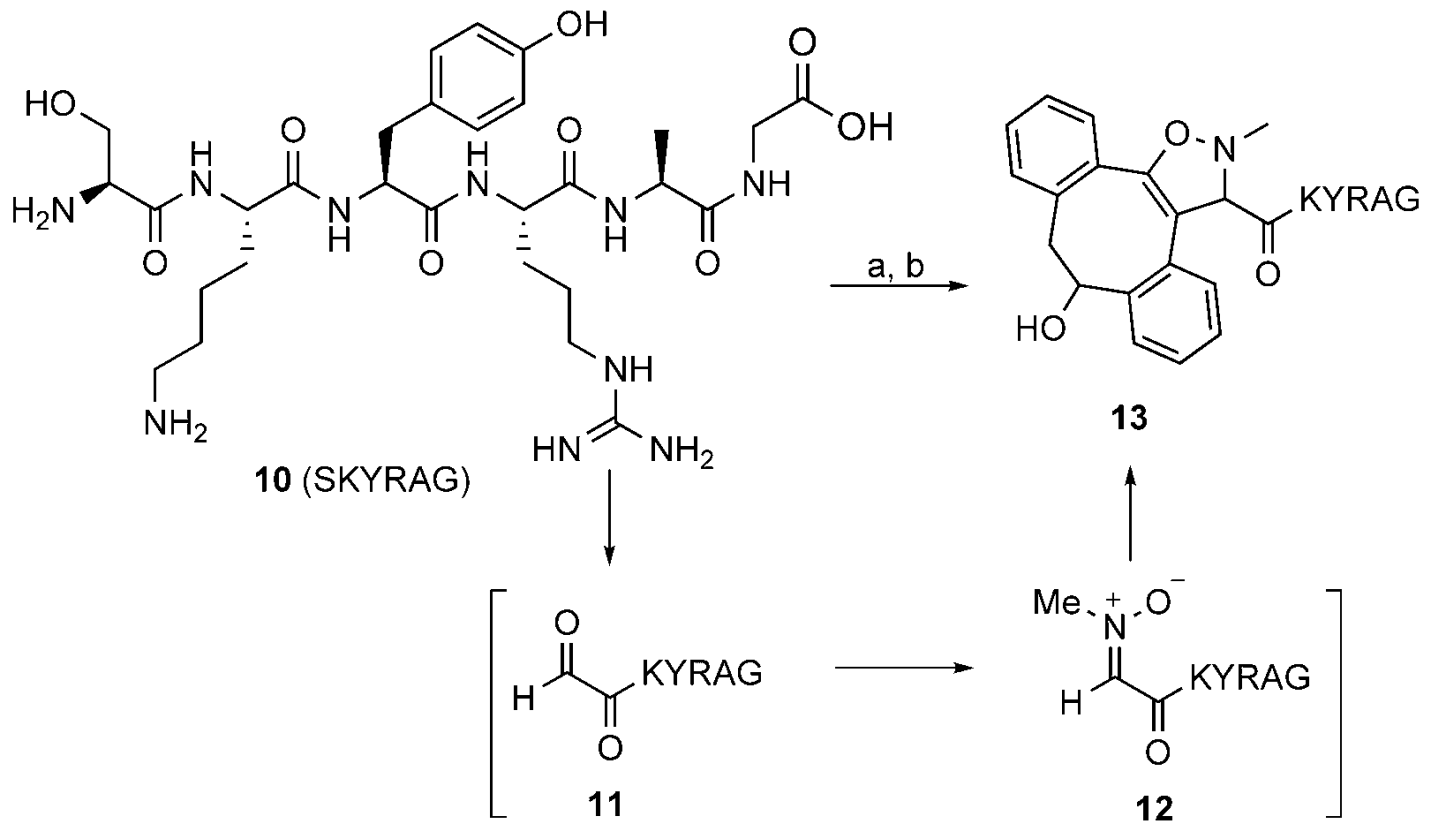
One-pot N-terminal conjugation of a hexapeptide by SPANC: a) 1. NaIO_4_, NH_4_OAc buffer, pH 6.8, room temperature, 1 h; 2. *p*-MeOC_6_H_4_SH, room temperature, 1 h; then *p*-MeOC_6_H_4_NH_2_, MeHNOH⋅HCl, room temperature, 20 min; b) **2**, room temperature, 1 h.

Fortunately, SPANC could be employed for efficient peptide and protein modification by implementing a one-pot three-step procedure. Thus, the N-terminal serine residue of model peptide **10** was oxidized^20^ with sodium periodate (1.1 equiv) to rapidly generate aldehyde **11**, which was first treated with *p*-methoxybenzenethiol (6.6 equiv, 30 min), and then with *N*-methylhydroxylamine (2.2 equiv), *p-*anisidine (5 equiv), and **2** (2.2 equiv) to give the desired isoxazoline **13** via nitrone **12** (Scheme 1). We found that treatment with *p*-MeOC_6_H_4_SH was essential to avoid the conversion of *N*-methylhydroxylamine into nitrosomethane dimer ((MeNO)_2_) by oxidation with iodate (IO_3_^−^) formed in the previous step.^21^ Furthermore, the rate of nitrone formation was greatly enhanced by the addition of *p-*anisidine, probably by a similar mechanism to that described for the formation of oximes from aldehydes and hydroxylamines.^22^

To examine whether the one-pot three-step protocol was suitable for protein modification, we selected the chemokine interleukin-8 (IL-8),^23^ as this prototypical protein has an N*-*terminal serine residue and a relatively low molecular weight (72 amino acids, MW=8382 Da), which facilitates direct analysis of chemical modification by mass spectrometry. Current labeling methods of IL-8, for example, for the installment of a radiolabel for scintigraphic imaging of infections,^24^ are based on random reactions of side-chain lysine amino groups with no control over the number of reactions that take place or the sites of reaction.

**Figure 3:**
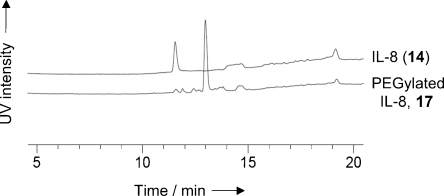
HPLC traces of IL-8 (**14**) and crude PEGylated IL-8, **17**.

**Scheme 2:**
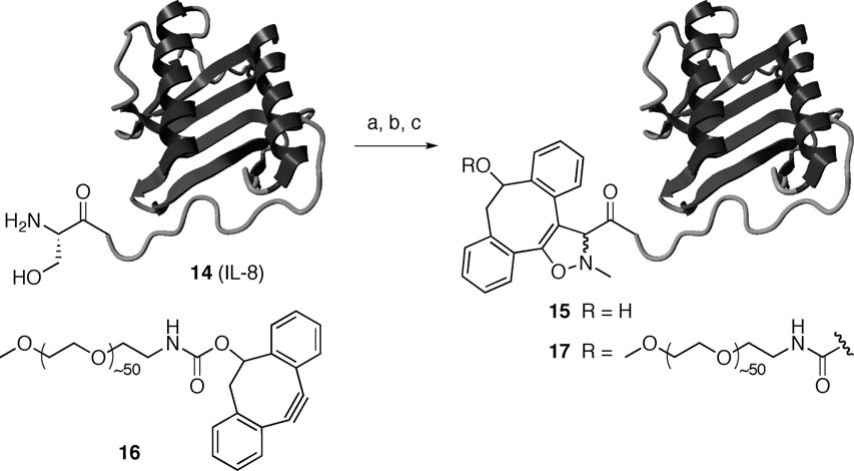
One-pot N-terminal functionalization of IL-8 by SPANC: a) 1. NaIO_4_, NH_4_OAc buffer, pH 6.9, room temperature, 1 h; 2. *p*-MeOC_6_H_4_SH, room temperature, 2 h; b) *p*-MeOC_6_H_4_NH_2_, MeNHOH⋅HCl, room temperature, 20 min; c) cyclooctynol **2** or PEG–cyclooctyne **16**, room temperature, 20 h.

Thus, IL-8 in NH_4_OAc buffer (2 mm, pH 6.9) was subjected to oxidation with NaIO_4_ (1.1 equiv, 1 h), followed by treatment with *p*-MeOC_6_H_4_SH (6.6 equiv, 2 h), then *N*-methylhydroxylamine (10 equiv) and *p-*anisidine (10 equiv), and finally cyclooctynol **2** (25 equiv, 21 mm). After 24 h, mass spectrometric analysis showed the presence of a single protein with a mass corresponding to the isoxazoline conjugate **15** (MW=8599 Da; Scheme 2). The one-pot three-step SPANC protocol was also successfully employed to PEGylate^25^, ^26^ IL-8 by using the PEG_2000_-modified dibenzocyclooctyne **16** (PEG=poly(ethylene glycol)). Quantitative formation of PEG-modified IL-8 **17** was observed by HPLC analysis (Figure 3).

We have shown that 1,3-dipolar cycloadditions of cyclooctynes with nitrones that contain ester or amide α substituents exhibit much faster kinetics than similar reactions with azides.^27^ The new methodology was successfully employed for the site-specific modification of a peptide and a protein by implementing a one-pot three-step protocol. Besides serine or threonine oxidation, a variety of methods have been described for the installment of carbonyl groups in proteins,^28^ and it is to be expected that SPANC is compatible with these approaches. Furthermore, metal-free click reactions have found entry into materials science.^11^ SPANC will provide an additional tool for the preparation of increasingly complex materials by simple and flexible chemical manipulations. Finally, we anticipate that SPANC will offer an attractive alternative to the well-established oxime ligation^29^ because the synthesis of nitrones is simple,^19^ the isoxazoline products are stable,^14^ and the combination of a functionalized nitrone (R^3^ is a functional group, Table 1) with a cyclooctyne conjugate (such as **16**) will make it possible to introduce two different functionalities in a single process.
