# Supplementary material for: Protein Modification by Strain-Promoted Alkyne–Nitrone Cycloaddition
Source: Angew Chem Int Ed Engl. 2010 Mar 23;49(17):3065–8. doi: 10.1002/anie.201000408 (PMC2871956; doi:10.1002/anie.201000408)
Supplement: Supplementary file 1 [file anie_3065_SD1.pdf]

Angewandte Chemie
Eine Zeitschrift der Gesellschaft Deutscher Chemiker

Supporting Information

© Wiley-VCH 2010

69451 Weinheim, Germany

Protein Modification by Strain-Promoted Alkyne–Nitrone Cycloaddition**

Xinghai Ning, Rinske P. Temming, Jan Dommerholt, Jun Guo, Daniel B. Ania, Marjoke F. Debets, Margreet A. Wolfert, Geert-Jan Boons, and Floris L. van Delft**

anie_201000408_sm_miscellaneous_information.pdf

General methods and procedures

^1H NMR spectra were recorded in $CDCl_3$ or D_2O on Varian Merc-300 or Varian Inova-500 spectrometers equipped with Sun workstations at 300 K. TMS (δ_H 0.00) or D_2O (δ_H 4.67) was used as the internal reference. ^{13}C NMR spectra were recorded in $CDCl_3$ or D_2O at 75 MHz on a Varian Merc-300 spectrometer, respectively, using the central resonance of $CDCl_3$ (δ_C 77.0) as the internal reference. COSY, HSQC, HMBC and TOCSY experiments were used to assist signal assignment of the spectra. Mass spectra were obtained on Applied Biosystems Voyager DE-Pro MALDI-TOF (no calibration), Bruker Daltonics 9.4T (FTICR, external calibration with BSA) or JEOL AccuToF. Optical rotatory power was obtained on Jasco P-1020 polarimeter at 300 K. Chemicals were purchased from Aldrich or Fluka and used without further purification. CH_2Cl_2, acetonitrile and toluene were distilled from calcium hydride; THF from sodium; and CH_3OH from magnesium and iodine. Mariculture keyhole limpet hemocyanin (mcKLH), maleimide activated bovine serum albumin (BSA-MI), and succinimidyl 3-(bromoacetamido)propionate (SBAP) were purchased from Pierce Endogen, Rockford, IL. Aqueous solutions are saturated unless otherwise specified. All reactions were performed under anhydrous conditions under argon and monitored by TLC on Kieselgel 60 F254 (Merck). Detection was by examination under UV light (254 nm) and by charring with 10% sulfuric acid in methanol. Silica gel (Merck, 70-230 mesh) was used for chromatography. Analytical reversed-phase high performance liquid chromatography (RP-HPLC) was performed on a Shimadzu LC-2010AHT HPLC using an analytical reversed-phase RPC18 column (Varian intersil ODS-3, 100 Å, 5 µm, 150 mm x 4.6 mm) and a Shimadzu UV detector operating at 254 nm. Elution was effected using a gradient of 10-70% of CH_3CN in water (both containing 0.1% TFA) over 30 minutes at a flow rate of 1.0 mL/min.

*Procedures for preparation and analytical data of nitrones **4a**-**f**.*

Nitrones **4a**,**b**,**d**-**f** were prepared according to the method described by Parhi *et al.*[1] Nitrone **4c** was prepared according to Tian *et al.*[2]

Nitrones **4a**[3], **4b**[4], **4c**[2] and **4e**[3] are known compounds. Analytical data for nitrones **4d** and **4f** are given below.

Ethyl (1-(*N*-methylimino)ethyl *N*-oxide)propanoate (4d)

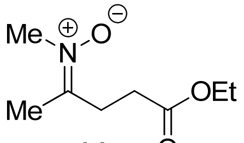

4d

Mixture of E/Z isomers.

^{1}H NMR ($CDCl_3$, 400 MHz): δ 4.03-4.12 (m, 2H), 3.71 (s, 1.2H), 3.62 (s, 2.8H), 2.64-2.71 (m, 3H), 2.45-2.51 (m, 1H), 2.08 and 2.05 (2x s, 3H), 1.19 (q, 3H). ^{13}C NMR ($CDCl_3$, 75 MHz): δ 172.8, 171.3, 146.0, 146.0, 87.4, 61.0, 60.5, 47.9, 47.3, 44.1, 37.8, 30.8, 29.2, 28.7, 28.4, 19.8, 17.4, 14.1, 14.0. HRMS (ESI+) *m/z* calcd for $C_8H_{16}NO_3$ $(M + H)^+$: 174.1130, found: 174.1122.

***N*-(Methyliminoacetyl *N*-oxide)benzylamine (4f)**

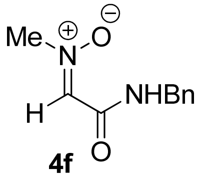

4f

^{1}H NMR ($CDCl_3$, 400 MHz): δ 10.12 (bs, 1H), 7.32 (bs, 5H), 7.12 (s, 1H), 4.56 (d, 2H), 3.81 (s, 3H). ^{13}C NMR ($CDCl_3$, 75 MHz): δ 160.3, 137.6, 131.9, 128.6, 127.6, 127.4, 54.5, 42.8. HRMS (ESI+) *m/z* calcd for $C_{10}H_{13}N_2O_2$ $(M + H)^+$: 193.0977, found: 193.0969.

*General procedure for cycloaddition of **2** and **4a-f** leading to isoxazolines **5a-f**.*

To a solution of **2** (0.1 mmol) in a 1:1 mixture of CH_3CN and H_2O (2 mL) was added **4a-f** (0.1 mmol). The mixture was stirred at room temperature until TLC analysis indicated complete consumption of starting materials. The reaction mixture was concentrated *in vacuo*, and the residue was purified by column chromatography on silica gel.

2-Methyl-3-phenyl-2,3,8,9-tetrahydrodibenzo[3,4:7,8]cycloocta[1,2-d]isoxazol-8-ol (5a)

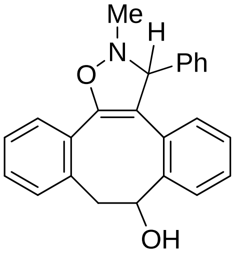

5a

Prepared according to the general procedure by stirring for 16 h.

R_F 0.3 (EtOAc:heptane, 1:2). Yield 95%, isolated as a mixture of regio- and diastereomers.

^{1}H NMR ($CDCl_3$, 400 MHz): δ 6.84-7.66 (m, 13H), 5.26 (dd, 0.5H), 5.01-5.14 (m, 1.5H), 3.67(dd, 0.3H), 3.33-3.42 (m, 0.3H), 3.06-3.23 (m, 4.4H). ^{13}C NMR ($CDCl_3$, 75 MHz): δ 146.8, 144.1, 140.6, 140.5, 140.2, 136.9, 136.4, 132.6, 131.4, 129.7, 128.6, 128.5, 127.2, 127.1, 126.7, 126.3, 111.2, 110.0, 80.7, 80.4, 73.7, 68.7, 47.2, 46.8, 45.3, 42.8, 41.8, 40.1. HRMS (ESI+) *m/z* calcd for $C_{24}H_{22}NO_2$ $(M + H)^+$: 356.1651, found: 356.1637.

2-Methyl-3-phenethyl-2,3,8,9-tetrahydrodibenzo[3,4:7,8]cycloocta[1,2-d]isoxazol-8-ol (5b)

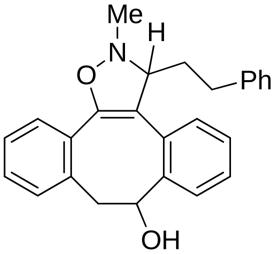

5b

Prepared according to the general procedure by stirring for 16 h.

R_F 0.3 (EtOAc:heptane, 1:3). Yield 80%, isolated as a mixture of regio- and diastereomers.

^{1}H NMR ($CDCl_3$, 400 MHz): δ 7.07-7.69 (m, 13H), 5.69 (dd, 0.3H), 5.47-5.55 (m, 0.3H), 5.14 (dd, 0.2H), 5.04 (dd, 0.3H), 4.14 (dd, 0.2H), 3.95-4.04 (0.8H), 3.67-3.81 (m, 0.6H), 3.50-3.62 (m, 0.6H), 3.25-3.36 (m, 0.6H), 3.13-3.21 (m, 0.6H), 2.96, 2.95 (2x s, 3H), 2.54-2.94 (m, 2.3H), 2.25-2.39 (m,

0.5H, OH), 1.72-2.22 (m, 2.7H), 1.58-1.68 (m, 0.4H, OH). ^{13}C NMR ($CDCl_3$, 75 MHz): δ 148.5, 146.4, 143.9, 141.8, 141.7, 141.6, 140.3, 140.1, 137.3, 137.2, 136.5, 136.1, 133.1, 131.4, 130.3, 130.0, 129.6, 128.8, 128.5, 128.4, 128.3, 127.6, 127.3, 127.2, 126.8, 126.4, 125.8, 125.7, 125.4, 112.6, 111.2, 110.9, 109.7, 76.1, 74.3, 71.3, 69.0, 47.2, 47.0, 46.8, 46.7, 45.6, 42.9, 41.5, 35.6, 32.6, 32.4, 32.3, 32.2. HRMS (ESI+) *m/z* calcd for $C_{26}H_{26}NO_2$ $(M + H)^+$: 384.1964, found: 384.1943.

2,3-Diphenyl-2,3,8,9-tetrahydrodibenzo[3,4:7,8]cycloocta[1,2-d]isoxazol-8-ol (5c)

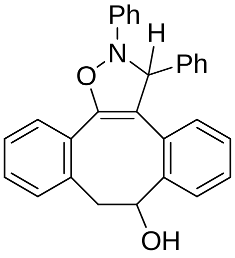

5c

Prepared according to the general procedure by stirring for 16 h.

R_F 0.4 (EtOAc: heptane, 1:2). Yield 89%, isolated as a mixture of regio- and diastereomers.

^{1}H NMR ($CDCl_3$, 400 MHz): δ 7.74-7.82 (m, 1.6H), 6.56-7.57 (m, 20H), 6.22-6.30 (m, 0.3H), 5.89-5.99 (m, 0.3H), 5.61-5.70 (m, 0.5H), 5.40 (s, 0.3H), 5.27 (s, 0.5H), 5.12-5.17 (m, 0.4H), 4.57 (s, 0.3H), 3.90-4.02 (m, 0.7H), 3.66-3.71 (0.3H), 3.48-3.59 (m, 0.4H), 3.28-3.42 (m, 0.5H), 2.76-2.96 (m, 0.6H), 2.15 (bs, 0.3H), 1.92 (bs, 0.3H), 1.55 (bs, 0.4H). ^{13}C NMR ($CDCl_3$, 75 MHz): δ 147.3, 147.0, 146.4, 143.2, 141.0, 138.9, 135.5, 133.0, 131.9, 130.2, 129.8, 129.1, 128.9, 128.3, 128.2, 127.9, 127.1, 126.5, 126.2, 123.0, 121.6, 121.3, 120.8, 118.6, 117.9, 117.7, 115.8, 98.3, 98.2, 93.4, 73.7, 70.0, 49.3, 45.0, 44.3, 41.2, 41.1, 36.6, 35.5. HRMS (ESI+) *m/z* calcd for $C_{29}H_{24}NO_2$ $(M + H)^+$: 418.1807, found: 418.1795.

Ethyl 3-(8-hydroxy-2,3-dimethyl-2,3,8,9-tetrahydrodibenzo[3,4:7,8]cycloocta[1,2-d]isoxazol-3-yl)propanoate (5d)

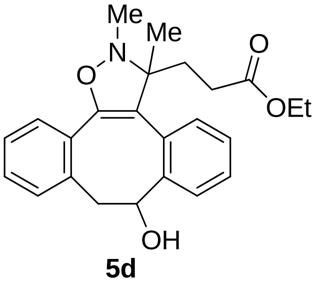

5d

Prepared according to the general procedure by stirring for 72 h.

R_F 0.3 (EtOAc: heptane, 1:1). Yield 33%, isolated as a mixture of regio- and diastereomers (contaminated with uncharacterized side-products), main peaks are given.

^{1}H NMR ($CDCl_3$, 400 MHz): δ 6.90-7.73 (m, 8H), 5.76-5.87 (m, 0.2H), 5.54 (d, 0.3H), 5.43-5.46 (m, 0.4H), 4.03-4.20 (m, 2H), 3.85 (d, 0.2H), 3.70-3.75 (m, 0.4H), 3.58 (dt, 0.6H), 3.32 (dd, 0.8H), 2.96-3.14 (m, 1.4H), 2.86 (s, 1.1H), 2.84 (s, 0.3H), 2.71-2.80 (m, 0.9H), 2.03-2.31 (m, 2H), 1.43 (s, 0.4H), 1.18-1.42 (m, 3H). ^{13}C NMR ($CDCl_3$, 75 MHz): δ 174.1, 154.5, 139.0, 131.5, 131.2, 128.8, 128.6, 127.8, 127.6, 126.9, 126.7, 126.1, 125.2, 124.7, 120.7, 120.4, 110.1, 109.6, 106.9, 95.3, 93.1, 86.7, 84.7, 83.5, 74.3, 73.1, 60.6, 60.4, 60.4, 50.2, 44.0, 43.5, 42.2, 40.7, 36.5, 35.9, 29.8, 14.8. HRMS (ESI+) *m/z* calcd for $C_{24}H_{28}NO_4$ $(M + H)^+$: 394.2018, found: 394.2001.

Ethyl 8-hydroxy-2-methyl-2,3,8,9-tetrahydrodibenzo[3,4:7,8]cycloocta[1,2-d]isoxazole-3-carboxylate (5e)

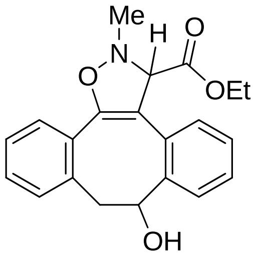

5e

Prepared according to the general procedure by stirring for 16 h.

R_F 0.4 (EtOAc: heptane, 2:1). Yield 92%, isolated as a mixture of regio- and diastereomers.

^{1}H NMR ($CDCl_3$, 400 MHz): δ 7.67-7.70 (m, 0.7H), 7.54-7.57 (m, 0.4H), 7.41-7.45 (m, 1H), 7.12-7.37 (m, 8.6H), 5.77-5.82 (m, 0.4H), 5.52-5.57 (m, 0.3H), 4.96-5.07 (m, 0.6H), 4.79 (s, 0.2H), 4.73 (s, 0.3H), 4.72 (s, 0.3H), 4.71 (s, 0.2H), 4.08-4.28 (m, 2.7H), 3.79-3.84 (m, 0.5H), 3.55-3.73 (m, 0.7H), 3.43 (dd, 0.2H), 3.13-3.29 (m, 1H), 3.09, 3.09, 3.08 (3x s, 2.4H), 1.25 (t, 0.3H), 1.20 (t, 0.6H), 1.15 (t, 1H), 1.11 (t, 0.9H). ^{13}C NMR ($CDCl_3$, 75 MHz): δ 169.7, 169.5, 151.2, 149.1, 144.2, 143.0, 140.5, 138.4, 137.4, 136.8, 133.1, 131.5, 130.2, 129.9, 129.7, 129.6, 129.3, 127.8, 127.7, 127.6, 127.4, 127.2, 126.7, 126.4, 125.7, 126.6, 105.3, 103.9, 78.4, 71.5, 68.6, 62.3, 61.5, 48.2, 47.5, 47.4, 47.3, 45.4, 42.6, 42.2, 40.2, 13.4. HRMS (ESI+) *m/z* calcd for $C_{21}H_{22}NO_4$ $(M + H)^+$: 352.1549, found: 352.1539.

***N*-benzyl-8-hydroxy-2-methyl-2,3,8,9-tetrahydrodibenzo[3,4:7,8]cycloocta[1,2-d]isoxazole-3-carboxamide (5f)**

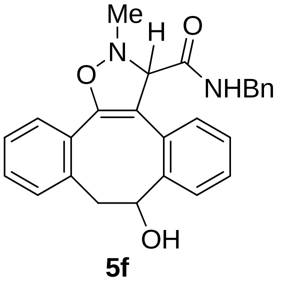

5f

Prepared according to the general procedure by stirring for 16 h.

R_F 0.4 (EtOAc: heptane, 2:1). Yield 93%, isolated as a mixture of regio- and diastereomers.

^{1}H NMR (CDCl$_3$, 400 MHz): δ 7.84 (t, 0.2H), 7.75 (t, 0.2H), 7.62-7.66 (m, 1H), 7.23-7.39 (m, 8.9H), 7.08-7.20 (m, 3.9H), 5.38 (dd, 0.2H), 5.23-5.29 (m, 0.5H), 4.98-5.03 (m, 0.3H), 4.86-4.95 (m, 0.3H), 4.65, 4.64 (2x s, 0.8H), 4.60 (s, 0.2H), 4.55 (dd, 0.3H), 4.48 (dd, 0.5H), 4.33-4.45 (m, 1.3H), 3.67 (dd, 0.2H), 3.54 (dd, 0.4H), 3.35 (dd, 0.2H), 3.08-3.27 (m, 1.3H), 3.05, 3.04, 3.03, 3.03 (4x s, 3H), 2.13-2.20 (m, 0.3H), 2.04-2.11 (m, 0.3H), 1.78 (bs, 0.5H). ^{13}C NMR (CDCl$_3$, 75 MHz): δ 169.2, 168.5, 168.3, 147.5, 146.8, 144.5, 143.0, 140.5, 139.1, 138.4, 138.3, 138.2, 137.7, 137.1, 136.8, 136.4, 133.1, 132.8, 131.8, 131.1, 129.8, 129.5, 129.4, 129.3, 128.6, 127.7, 127.5, 126.2, 125.6, 125.1, 109.0, 107.7, 107.6, 105.8, 78.5, 78.1, 77.9, 71.5, 68.4, 46.3, 46.2, 46.0, 45.3, 43.2, 43.0, 42.9, 42.7, 40.4. HRMS (ESI+) *m/z* calcd for $C_{26}H_{25}N_2O_3$ $(M + H)^+$: 413.1865, found: 413.1862.

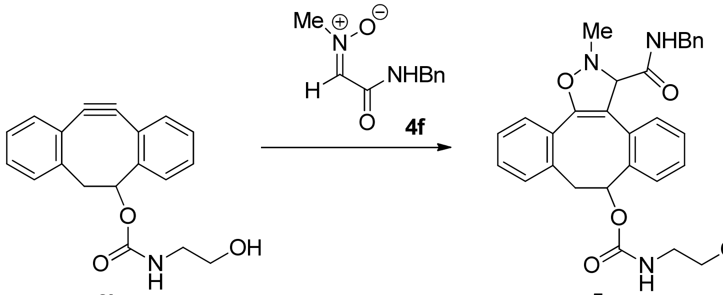

Prepared according to the general procedure by stirring **2b** and **4f** for 30 min.

Purified by column chromatography on silica gel (EtOAc:CH$_2$Cl$_2$, 1:1) led to the isolation of 4 isomers of **5g**. HRMS (ESI+) *m/z* calcd for $C_{29}H_{29}N_3O_5Na$ $[M+Na]^+$ 522.1999, found 522.2030. **Isomer 1**: R_F = 0.24 (EtOAc : CH$_2$Cl$_2$ 1 : 1). ^{1}H NMR (300 MHz, CDCl$_3$): δ 2.09 (bs, 1H), 3.06 (s, 3H), 3.18-3.37 (m, 4H), 3.70 (bs, 2H), 4.30-4.50 (m, 2H), 4.66 (s, 1H), 5.00 (bs, 1H), 6.12-6.18 (m, 1H), 7.18-7.63 (m, 13H). MS (LCQ): *m/z* calcd for $C_{29}H_{29}N_3O_5Na$ $[M+Na]^+$ 522.2, found 522.3.

Isomer 2: R_F = 0.17 (EtOAc : CH_2Cl_2 1 : 1). ^{1}H NMR (300 MHz, $CDCl_3$): δ 3.04 (s, 3H), 3.13-3.33 (m, 4H), 3.65 (bs, 2H), 4.35-4.41 (m, 2H), 4.67 (s, 1H), 5.13 (bs, 1H), 6.06-6.14 (m, 1H), 7.12-7.64 (m, 13H). MS (LCQ): *m/z* calcd for $C_{29}H_{29}N_3O_5Na$ $[M+Na]^+$ 522.2, found 522.5. **Isomers 3 + 4**. R_F = 0.13 and 0.10 (EtOAc : CH_2Cl_2 1 : 1). ^{1}H NMR (300 MHz, $CDCl_3$): δ 2.12 (bs, 1H), 3.04 (s, 3H), 3.07 (s, 3H), 3.17-3.56 (m, 10H), 3.73 (bs, 2H), 4.35-4.46 (m, 2H), 4.49-4.67 (m, 4H), 5.00 (bs, 1H), 5.18 (bs, 1H), 6.11-6.20 (m, 1H), 6.29-6.38 (m, 1H), 7.09-7.72 (m, 26H). MS (LCQ): *m/z* calcd for $C_{29}H_{29}N_3O_5Na$ $[M+Na]^+$ 522.2, found 522.3.

General procedure kinetic experiments

NMR experiments

Stock solution A: alkyne **2** (79 mg, 0.36 mmol) was dissolved in a mixture of CD_3CN and D_2O (ratio 3:1, 10 mL) to give a 36 mM solution.

Stock solution B (32.8 mM): nitrone **4a-d** (0.338 mmol) was dissolved in a mixture of CD_3CN and D_2O (ratio 3:1, 10 mL) to give a 32.8 mM solution.

^{1}H-NMR monitoring of cycloaddition of **2** and **4** was performed by rapid mixing (t=0) of stock solutions A and B (0.3 mL each) in an NMR tube and immediate insertion into a 400 MHz NMR spectrometer. NMR spectra were measured at preset time-intervals.

Kinetics of the reaction of **2** with nitrone **4a** (Table 1, entry 1) were determined by the measuring the decrease of the integral of the signal caused by the nitrone methyl groups, with the integral of the acetonitrile solvent-peak as internal standard. A starting value for the integral of the methyl signals was estimated, due to the fact that cycloaddition had already proceeded significantly by the time of the first measurement. The conversion was determined by dividing the combined integrals of the methyl groups of **5a** and **6a** by the estimated starting value.

For the reaction of **2** with nitrone **4b** (Table 1, entry 2), kinetics were determined by integration of the methyl peaks of the nitrone in starting material and product. The sum of these two integrals was taken as internal standard, due to the fact that the acetonitrile peak was not distinct from other signals. Now, conversion was determined by the relative increase of methyl signals of product **5** and **6**, compared to the sum of both signals.

The reaction of **2** with nitrone **4c** was too fast to follow by NMR. By the time of the first measurement, the reaction had already reached such a conversion that no reliable kinetic plot could be made. Therefore only a rough estimate was made based on the first measurement.

From the conversion plots thus obtained, the second order rate plots were calculated according to equation (1).

$$kt = \frac{1}{[B]_0-[A]_0} \times \ln \frac{[A]_0([B]_0-[P])}{([A]_0-[P])[B]_0} \qquad (1)$$

with k = 2nd order rate constant ($M^{-1}s^{-1}$), t = reaction time (s), $[A]_0$ = the initial concentration of substrate A (mmol/mL), $[B]_0$ = the initial concentration of substrate B (mmol/mL) and $[P]$ = the concentration of product (mmol/mL). As an example, the logarithmic plot of the reaction between nitrone **4b** and **2** is shown in Graph S1.

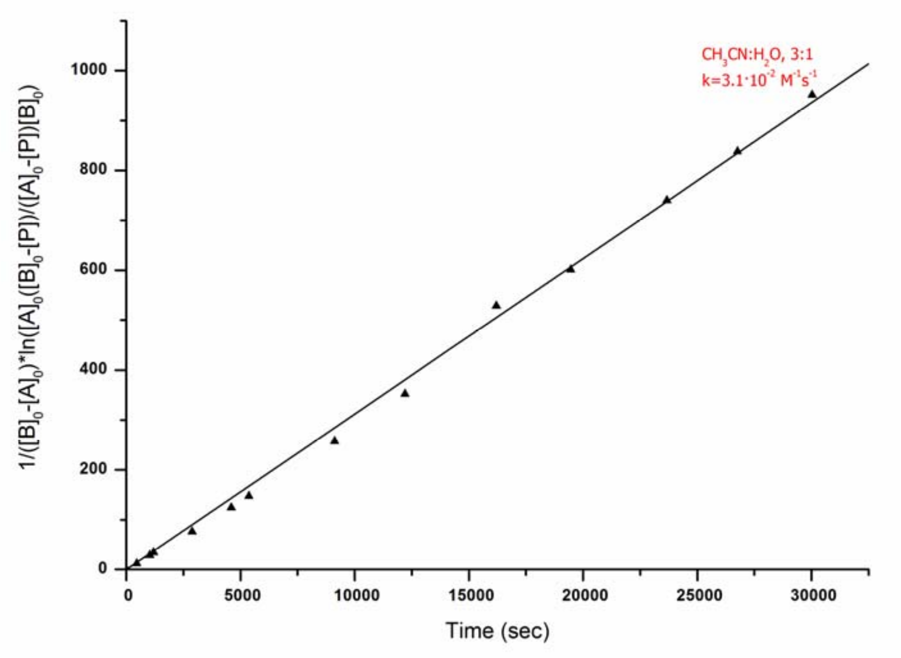

Graph S1. Logarithmic plot of the reaction of nitrone 4b with dibenzocyclooctyne 2.

UV-experiments

Stock solution A: alkyne **2**, **2b** or **3** (110 mg, 0.5 mmol) was dissolved in CH_3CN (50 mL) to give a 10 mM solution.

Stock solution B: nitrone **4e** or **f** (0.5 mmol) was dissolved in CH_3CN (50 mL) to give a 10 mM solution.

To achieve the desired CH_3CN:H_2O ratio, to 1 mL of stock solution A or B was added the correct amount of H_2O, giving solutions A' and B'. Solutions A' and B' were left to adjust to room temperature for at least 30 minutes.

UV monitoring of cycloaddition of dibenzocyclooctyne (**2** or **3**) and nitrone **4** was performed by rapid mixing (t=0) of solutions A' and B' (1 mL each) and immediate insertion into a 1 mm UV-cuvette.

Next, UV-absorption was measured at preset time intervals (in general every five seconds) at 304 nm. The graphs thus obtained showed a decrease of the absorption over time, sloping to a final absorption that was subtracted from the measured absorptions to obtain a relative absorption. To determine conversion, the relative absorption was divided by the difference between the maximal and the minimal absorption. Since the reactions already proceeded before measurement of the first absorption, the maximum absorption was adjusted to an estimated starting point.

From the conversion plots thus obtained, the second order rate plots were calculated according to equation (1).

In Graph S2 the logarithmic plot of nitrone **4e** with dibenzocyclooctyne **2** is shown.

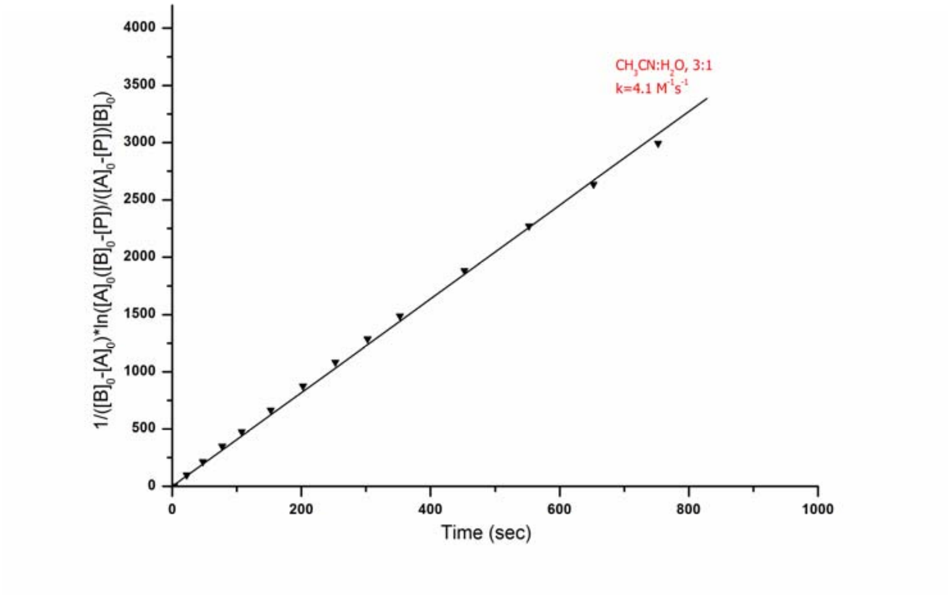

Graph S2. Logarithmic plot of the reaction between dibenzocyclooctyne 2 and nitrone 4e.

Table 1. Rate constants for the reaction of dibenzocyclooctyne **2** or **2b** with nitrone **4f**.

entry	cyclooctyne	[cyclooctyne] (mM)	[**4f**] (mM)	ratio $CH_3CN:H_2O$	k (M^{-1} s^{-1})
1	**2**	1.00	1.00	1:1	2.7
2	**2b**	1.00	1.20	1:1	2.2
3		0.33	0.30	1:1	2.3
4		0.33	0.30	1:3	5.5
5		0.33	0.30	1:5	8.8
6		0.33	0.30	1:9	12.8

In Graph S3 the logarithmic plots of the reaction of nitrone **4f** with dibenzocyclooctyne **2b**[5] in different solvent mixtures is visualized. The graph clearly shows an increase of reaction rate upon increase of the polarity of the solvent mixture. When the reaction was performed in the same solvent mixture but at different concentration and with a different ratio between the two reactants (Table 2, entry 2 and 3), identical logarithmic plots were obtained.

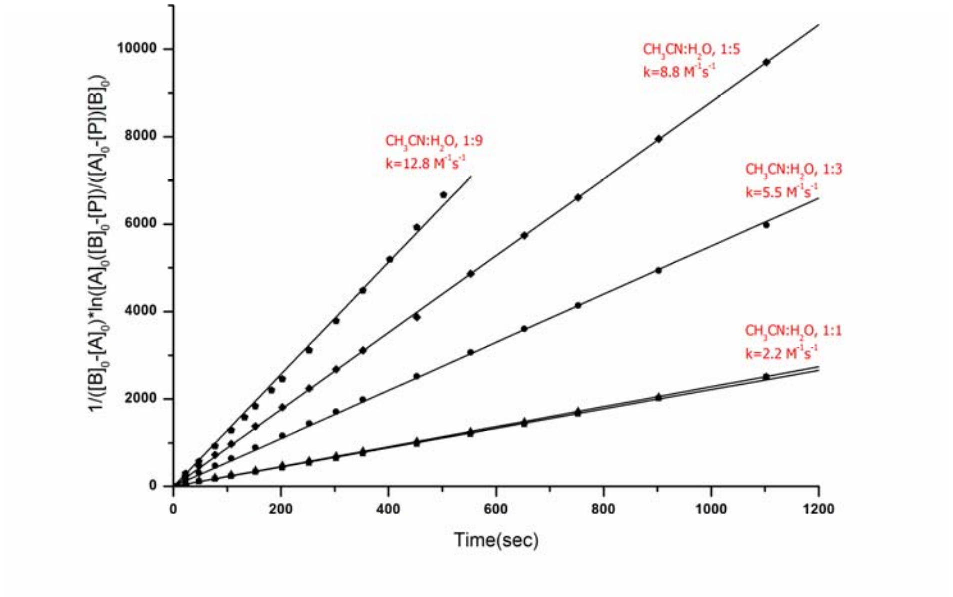

Graph S3. Logarithmic plot of nitrone 4f with dibenzocycooctyne 2b in different solvent mixtures.

Table 2. Rate constants for the reaction of azadibenzocyclooctyne **3** with nitrone **4f**.

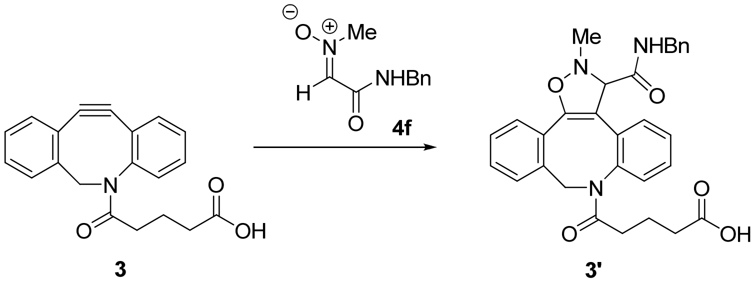

Entry	cyclooctyne	[cyclooctyne] (mM)	[**4f**] (mM)	ratio $CH_3CN:H_2O$	k (M^{-1} s^{-1})
1	**3**	0.33	0.30	3:1	8.5
2		0.33	0.30	1:3	39

HRMS (ESI+) *m/z* calcd for **3'** $C_{30}H_{29}N_3O_5Na$ $[M+Na]^+$: 534.2005, found 534.2002.

Synthesis of nitrone sugars

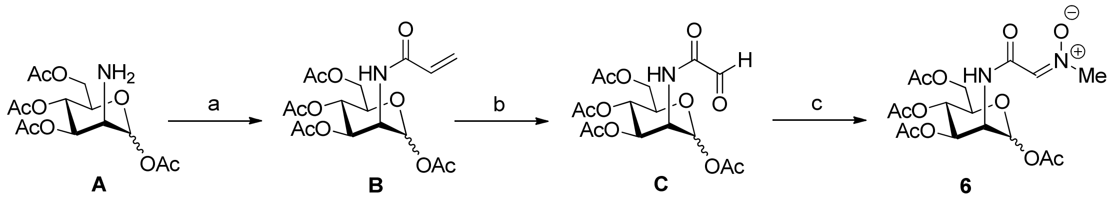

Scheme 1. Reagents and conditions (a) CH_2=CHC(O)Cl, *i*-Pr_2EtN, CH_2Cl_2 (62%); (b) OsO_4, $NaIO_4$, *t*-BuOH, H_2O, (c) MeHNOH.HCl, $NaHCO_3$, Et_2O, H_2O (46% for three steps).

1,3,4,6-Tetra-*O*-acetyl-*N*-(methyliminoacetyl *N*-oxide)-β-D-mannosamine pyranose (6)

To a solution of **A**[6] (39 mg, 0.11 mmol) in CH_2Cl_2 (3 mL) at 0 °C was added *i*-Pr_2EtN (48 µL, 0.28 mmol) and dropwise acryloyl chloride (1 mL from a stock solution of 90 µL in 5 mL CH_2Cl_2 (0.17 mmol) sequentially and the mixture was stirred at 0 °C for 2 h, when TLC chromatography indicated the complete conversion of **A** into a more lipophilic product (EtOAc, R_F 0.8). The reaction mixture was quenched with saturated $NaHCO_3$ (1 mL), and the layers were separated. The organic layer was dried ($MgSO_4$), filtered and concentrated *in vacuo* in the dark at 20 °C to give compound **B**. Without further purification and in the dark, crude compound **B** was dissolved in a 3:1 mixture of 1,4-dioxane:H_2O (400 µL), and solid $NaIO_4$ (72 mg, 0.33 mmol) was added. followed by a stock solution of OsO_4 in *t*-BuOH (2.5% solution, 9 µL). The solution was stirred rigorously for 16 h, until TLC chromatography indicated the complete conversion of **B** into a more hydrophilic product (EtOAc, R_F 0.2). The reaction mixture was diluted with MeOH (3 mL), filtered over Celite and concentrated. Rapid elution over a pad of silica gel (EtOAc/heptane, 6/1) and concentration gave crude **C** as an oil (35 mg, 0.087 mmol). Crude **C** was immediately dissolved in a 1:1 mixture of diethyl ether and H_2O (1 mL) . To the solution was added *N*-methylhydroxylamine hydrochloride (7 mg, 0.11 mmol) and $NaHCO_3$ (23 mg, 0.27 mmole). The mixture was stirred at room temperature for 30 min and after this time TLC analysis indicated the formation of two products (EtOAc, R_F 0.2 and 0.1). The reaction mixture was filtered and the mixture was extracted with EtOAc (3 × 3 mL). The organic combined layers were washed with H_2O (1 mL) and brine (1 mL), before drying ($MgSO_4$), filtration and concentration. The oily residue was purified by column chromatography on silica gel (eluent: EtOAc). Compound **6** (22 mg, 46% for three steps) eluted first, as a 6:1 mixture of anomeric acetates. MALDI HRMS: m/z 455.2220 [M + Na^+]. Calcd for $C_{17}H_{24}N_2O_{11}Na^+$ 455.1273. ^1H NMR (300 MHz, $CDCl_3$, major α-anomer shown): δ 7.04 (s, 1H), 6.02 (d, 1H, *J* = 1.8 Hz), 5.31

(dd, 1H, J = 5.1, 9.0 Hz), 5.27 (t, 1H, J = 9.3 Hz), 4.66 (ddd, 1H, J = 2.1, 3.6, 9.1 Hz), 4.10-4.23 (m, 2H), 3.93-3.99 (m, 1H), 3.77 (s, 3H), 2.11, 2.09, 1.99, 1.94 (4 × s, 12H).

The more polar compound eluted next (8 mg, 18% for three steps). Mass spectral analysis by MALDI HRMS showed mass peaks of 413.2127 [M + Na$^+$] and 429.1974 [M + K$^+$], indicative of a molecular mass of 390.13, corresponding to **6** with loss of one acetyl group.

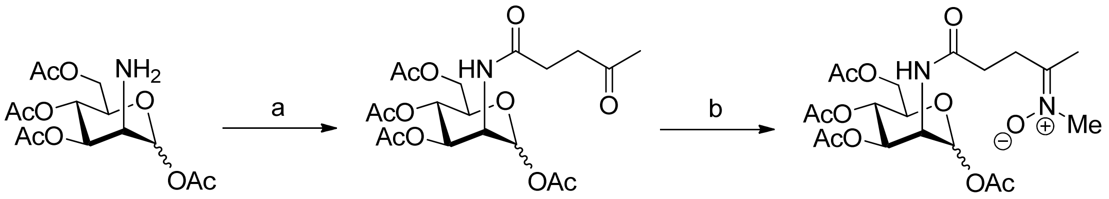

A **D** **7**

Scheme 2. Reagents and conditions (a) Levulinic anhydride, Et_3N, CH_2Cl_2 (61%); (b) MeHNOH.HCl, $NaHCO_3$, Et_2O, H_2O (56%).

1,3,4,6-Tetra-*O*-acetyl-*N*-(4-oxopentanoyl)-β-D-mannosamine pyranose (D)

To a solution of **A** (35 mg, 0.1 mmol) in CH_2Cl_2 (10 mL) was added levulinic anhydride[7] (42 mg, 0.2 mmol) and Et_3N (20 mg, 0.2 mmol). The mixture was stirred at rt for 12 h. The reaction mixture was concentrated *in vacuo*, and residue was purified by column chromatography on silica gel (eluant: hexane: EtOAc 1:3) to give **D**[8] (27 mg, 61%). ^{1}H NMR (300 MHz, $CDCl_3$): δ 6.75 (d, 1H, J = 9.3 Hz), 6.50 (d, 1H, J = 9.4 Hz), 5.87 (d, 1H, J = 1.9 Hz), 5.79 (d, 1H, J = 1.7 Hz), 5.19 (m, 1H), 4.95-5.10 (m, 2H), 4.66 (m, 1H), 4.49 (m, 1H), 4.10-4.23 (m, 2H), 3.93-4.05 (m, 4H), 3.68-3.77 (m, 1H), 2.65-2.73 (m, 4H), 2.33-2.55 (m, 4H), 1.88-2.06 (m, 30H).

1,3,4,6-Tetra-*O*-acetyl-*N*-(4-methyliminopentanoyl *N*-oxide)-β-D-mannosamine pyranose (7)

To a solution of **D** (22 mg, 0.05 mmol) in a mixture of Et_2O (10 mL) and H_2O (2 mL) was added *N*-methylhydroxylamine hydrochloride (41 mg, 0.1 mmol) and $NaHCO_3$ (42 mg, 0.1 mmol). The mixture was stirred at room temperature for 4h and after this time the reaction mixture was filtered and the mixture was evaporated *in vacuo*. The residue was purified by column chromatography on silica gel (eluent: CH_2Cl_2/CH_3OH 20:1) to give **7** (13 mg, 56%). ^{1}H NMR (300 MHz, $CDCl_3$): δ 5.92 (d, 1H, J = 2.1 Hz, H-1), 5.29 (m, 1H, H-3), 5.08 (m, 1H, H-5), 4.79 (s, 3H, NCH_3), 4.25 (m, 1H, H-2), 4.06-3.98 (m, 2H, H-6), 2.11-19.3 (m, 17H, 5Ac, CH_2-C=O). MALDI HRMS: m/z 497.1759 [M + Na$^+$]. Calcd for $C_{20}H_{30}N_2O_{11}Na^+$ 497.1742.

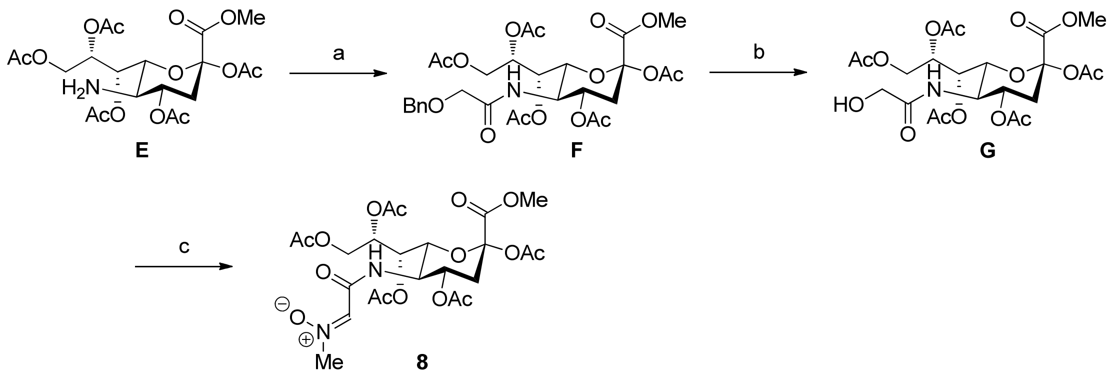

Scheme 3. Reagents and conditions (a) Benzyloxyacetyl chloride, Et_3N, CH_2Cl_2 (65%); (b) Pd-C, H_2, EtOH (86%); (c) *i.* Dess-Martin periodinane, CH_2Cl_2, *ii.* MeHNOH.HCl, $NaHCO_3$, Et_2O, H_2O (39%).

2,4,7,8,9-Penta-*O*-acetyl-*N*-(benzyloxyacetyl)-α-D-neuraminic acid methyl ester (F)

To the solution of **E**[9] (50 mg, 0.1 mmol) in CH_2Cl_2 (15 mL) was added benzyloxyacetyl chloride (39 mg, 0.2 mmol) and Et_3N (20 mg, 0.2 mmol) at 0 °C sequently. The mixture was stirred at 0 °C for 2 h. The reaction mixture was concentrated *in vacuo*, and residue was purified by column chromatography on silica gel (eluant: hexane: EtOAc 1:3) to give **F** (41 mg, 65%). ^{1}H NMR (300 MHz, $CDCl_3$): δ 7.35-7.16 (m, 5H, aromatic), 6.25 (m, 1H, NH), 5.39 (m, 1H, N*H*), 5.25 (m, 1H, H-6), 4.85 (m, 1H, H-7), 6.63 (s, 2H, C*H*$_2$OBn), 4.30-3.98 (m, 5H, H-9, H-8, H-5, H-4), 3.86 (m, 1H, H-3'), 3.72 (s, 3H, OC*H*$_3$), 3.08 (m, 1H, H-3), 2.09-1.86 (5 × s, 15H, 5Ac).^{13}C NMR (75 MHz, $CDCl_3$): *δ* 171.3, 171.2, 170.7, 170.5, 170.4, 170.0, 168.8, 166.8, 137.7, 129.3, 128.7, 128.6, 128.3, 97.3, 76.8, 73.8, 73.6, 72.9, 72.8, 72.3, 68.6, 67.6, 49.4, 38.6, 23.3, 21.2, 21.1, 21.0, 20.9. MALDI HRMS: m/z 662.2075 [M + Na$^+$]. Calcd for $C_{29}H_{37}NO_{15}Na^+$ 662.2055.

2,4,7,8,9-Penta-*O*-acetyl-*N*-(hydroxyacetyl)-α-D-neuraminic acid methyl ester (G)

To the solution of **F** (40 mg, 0.06 mmol) in EtOH was added Pd-C (10 wt. % on activated carbon, 5 mg). After which H_2 was bubbled through the solution for 1h followed by stirring under an H_2 atmosphere for 12 h. The reaction mixture was filtered and the mixture was evaporated *in vacuo*. The residue was purified by column chromatography on silica gel (eluent: CH_2Cl_2: CH_3OH 15:1) to give **9** (30 mg, 86%). ^{1}H NMR (300 MHz, $CDCl_3$): δ 6.41 (m, 1H, NH), 5.90 (m, 1H, N*H*), 5.23-5.13 (m, 3H, H-6, C*H*$_2$OH), 4.93 (m, 1H, H-7), 4.34-4.01 (m, 5H, H-9, H-8, H-5, H-4), 3.89 (m, 1H, H-3'), 3.72 (s, 3H, OC*H*$_3$), 3.02 (m, 1H, H-3), 2.11-1.84 (5 × s, 15H, 5Ac).^{13}C NMR (75 MHz,

CDCl3): δ 173.4, 172.1, 171.6, 171.3, 170.9, 168.9, 166.8, 129.2, 97.3, 76.8, 72.5, 68.3, 61.4, 49.5, 38.8, 36.6, 29.9, 23.4, 23.3, 21.2, 21.1, 21.0. MALDI HRMS: m/z 572.1599 [M + Na^+]. Calcd for $C_{22}H_{31}NO_{15}Na^+$ 572.1586.

2,4,7,8,9-Penta-*O*-acetyl-*N*-(methyliminoacetyl *N*-oxide)-α-D-neuraminic acid methyl ester (8)

To a stirred solution of **G** (28 mg, 0.05 mmol) in anhydrous CH_2Cl_2 was added Dess–Martin periodinane reagent (30 mg, 0.075 mmol) at 0 °C. The reaction mixture was allowed to stir at ambient temperature for 2 h then quenched with saturated $Na_2S_2O_3$ solutions (10 mL), and left to stir for a further 30 min. The aqueous phase was then extracted with EtOAc (10 mL ×3) and the recombinant organic extracts washed with distilled water (2 × 5 mL), and brine (5 mL). Drying over anhydrous $MgSO_4$, filtered and the mixture was evaporated *in vacuo*. The residue was dissolved in a mixture of Et_2O (10 mL) and H_2O (2 mL). And then mixture was added *N*-methylhydroxylamine hydrochloride (8.3 mg, 1.0 mmol) and $NaHCO_3$ (16 mg, 0.2 mmol). The mixture was stirred at room temperature for 8h and after this time the reaction mixture was filtered and the mixture was evaporated in vacuo. The residue was purified by column chromatography on silica gel (eluent: CH_2Cl_2: CH_3OH 15:1) to give **8** (11 mg, 39%). ^{1}H NMR (300 MHz, $CDCl_3$): δ 6.75 (m, 1H, C*H*=N-O), 5.45 (m, 1H, NH), 5.23-5.15 (m, 2H, H-6, N*H*), 4.91 (m, 1H, H-7), 4.38-3.60 (m, 12H, H-9, H-8, H-5, H-4, H-3', OC*H*3, NC*H*3), 2.82 (m, 1H, H-3), 2.13-1.86 (5 × s, 15H, 5Ac). MALDI HRMS: m/z 599.1711 [M + Na^+]. Calcd for $C_{23}H_{32}N_2O_{15}Na^+$ 599.1695.

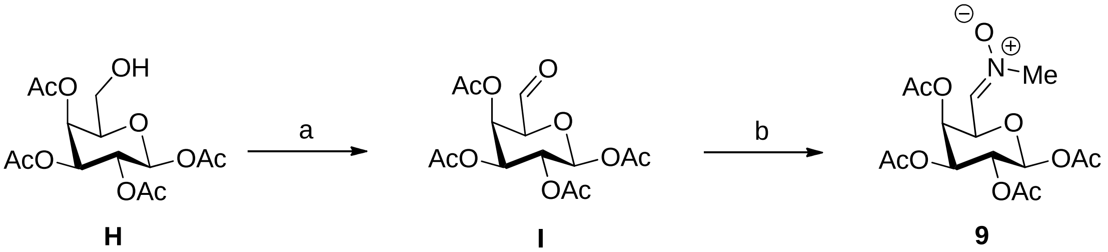

Scheme 4. Reagents and conditions (a) Dess-Martin periodinane, CH_2Cl_2 (81%), (b) MeHNOH.HCl, Et_3N, DCM (94%).

1,2,3,4-Tetra-*O*-acetyl-6-deoxy-6-aldo-β-D-galactopyranoside (I)

To a solution of 1,2,3,4-tetra-*O*-acetyl-β-D-galactopyranoside **H**[10] (0.210 g, 0.603 mmol) in DCM (10 ml) was added Dess-Martin reagent (0.307 g, 0.724 mmol) in one portion. The reaction mixture was stirred at RT for 2 h. The solution was filtered, washed with DCM, and concentrated. The mixture was purified by flash chromatography on silica (eluent: 1:1 EtOAc/hexanes) to give the product as colorless syrup which solidified upon standing (0.170 g, 81%). ^1H NMR (300 MHz, $CDCl_3$): δ 9.46 (s, 1H, H-6), 5.75 (m, 2H), 5.33 (t, 1H), 5.10 (dd, 1H), 4.31 (d, 1H), 2.12-1.93 (4 × s, 12H, 4Ac). ^{13}C NMR (75 MHz, $CDCl_3$): *δ* 195.6, 170.1, 169.8, 169.5, 169.3, 92.1, 78.0, 70.6, 67.6, 67.3, 20.9, 20.8, 20.7, 20.6. MALDI HRMS: m/z 369.0781 [M + Na^+]. Calcd for $C_{14}H_{18}O_{10}Na^+$ 369.0792.

1,2,3,4-Tetra-*O*-acetyl-6-deoxy-6-methyloxidoimino-β-D-galactopyranoside (9)

To a solution of **I** (42 mg, 0.121 mmol) in DCM (10 ml) was added *N*-hydroxylamine hydrochloride (10.1 mg, 0.121 mmol) and one drop of triethylamine. The reaction mixture was stirred at RT for 2 h. The solvent was evaporated and the residue was purified by flash chromatography on silica (eluent: EtOAc) to give **9** (43 mg, 94%). ^1H NMR (300 MHz, $CDCl_3$): δ 6.58 (d, 1H, C*H*=N-O), 5.68 (m, 2H), 5.28 (m, 1H), 5.16 (dd, 1H), 5.01 (d, 1H), 3.62 (s, 3H, NCH_3), 2.09-1.92 (4 × s, 12H, 4Ac). ^{13}C NMR (75 MHz, $CDCl_3$): δ 168.7, 168.5, 168.4, 167.8, 131.8, 91.1, 76.2, 69.6, 69.3, 66.9, 65.9, 51.7, 19.8, 19.7, 19.6, 19.5. MALDI HRMS: m/z 398.1072 [M + Na^+]. Calcd for $C_{15}H_{21}NO_{10}Na^+$ 398.1058.

Cell-surface labeling experiments[5]

Human Jurkat cells (Clone E6-1; ATCC) were cultured in RPMI 1640 medium (ATCC) with L-glutamine (2 mM), adjusted to contain sodium bicarbonate (1.5 g L^{-1}), glucose (4.5 g L^{-1}), HEPES (10 mM), and sodium pyruvate (1.0 mM) and supplemented with penicillin (100 u mL^{-1})/streptomycin (100 μg mL^{-1}; Mediatech) and fetal bovine serum (FBS, 10%; Hyclone). Cells were maintained in a humid 5% CO_2 atmosphere at 37 °C.

Jurkat cells were grown in the presence of compounds **6-9** (10, 20, 50 and 100 μM, final concentration) for 3 days. The metabolically labeled and untreated control cells were incubated with the biotinylated compound **J** (0-100 μM) in labeling buffer (DPBS, supplemented with FBS, 1%) for 0-180 min at room temperature. The cells were washed three times with labeling buffer and then incubated with avidin conjugated with fluorescein (Molecular Probes) for 15 min at 4 °C. Following three washes and cell lysis, cell lysates were analysed for fluorescence intensity (485 ex / 520 em) using a microplate reader (BMG, Labtech). Data points were collected in triplicate and are representative of three separate experiments. Cell viability was assessed at different points in the procedure with exclusion of trypan blue. No statistical significant increase in fluorescent intensity was observed for the metabolically labeled cells over control.

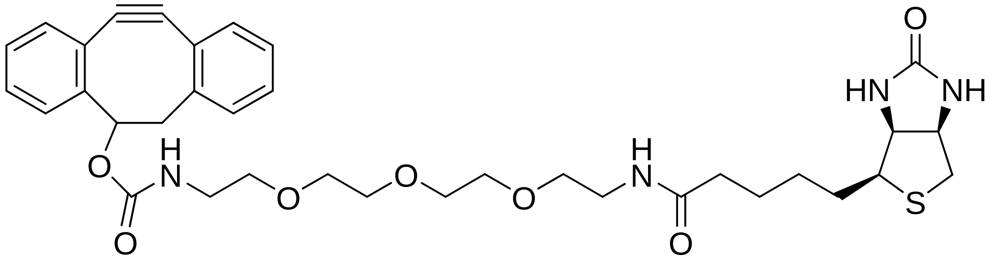

J

SKYRAG procedure

Oxazoline 13

Peptide **10** (SKYRAG, 5.5 mg, 8.08 μmol) was dissolved in 0.1 M NH_4OAc buffer (1 mL, pH 6.9), and $NaIO_4$ (1.9 mg, 8.89 μmol) was added. The resulting mixture was stirred at room temperature until mass spectral analysis (LCQ, 30 min) indicated oxidative cleavage was complete. Subsequently, 4-MeOPhSH (7.5 mg, 0.053 mmol) was added to give a white cloudy solution, and the new mixture was stirred for 1 h at room temperature, until the reaction mixture turned colourless. *p*-Anisidine (5.0 mg, 0.040 mmol) was then added and the solution was stirred until dissolved completely (15 min). N-methylhydroxylamine hydrochloride (1.5 mg, 0.018 mmol) and **2** (3.9 mg, 0.018 mmol) were added. The resulting reaction mixture was stirred at room temperature for 30 min to give isoxazoline **13**. HRMS (ESI +) *m/z* calcd for $C_{45}H_{59}N_{10}O_{10}$ $[M+H]^+$ 899.4410, found 899.4446.

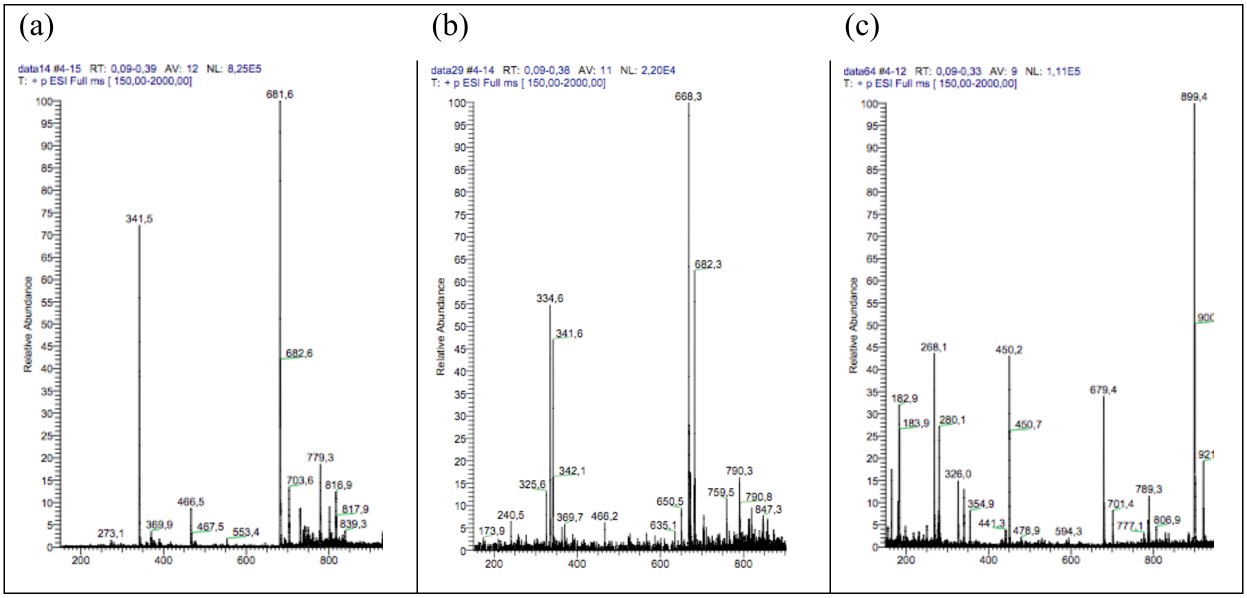

Figure 1. MS analysis of *N*-terminal SKYRAG conjugation by direct aliquoting from the reaction mixture into MeOH. (a) SKYRAG 10 in NH_4OAc buffer (20 mM, pH 6.9). (b) hydrate form of aldehyde-KYRAG **11** after $NaIO_4$ oxidation of **10**. (c) isoxazoline-KYRAG conjugate **13** after quench with *p*-MeOPhSH, then MeHNOH and **2**.

Labeling of IL-8

IL-8 labeling with cyclooctyne 2

A solution of IL-8[11] (5 µg, 0.60 nmol) in PBS buffer (10 µL) was diluted with NH_4OAc buffer pH 6.9 (10 µL). Subsequently, $NaIO_4$ (0.66 nmol, 0.14 µg) was added. The reaction was allowed to take place for 1 h at room temperature, and 4-MeOPhSH was added (3.9 nmol, 0.55 µg, 0.5 nL). After stirring for 1 h at room temperature, p-anisidine (5.6 nmol, 0.69 µg) was added. After stirring for 15 min at room temperature, N-methylhydroxylamine hydrochloride (5.6 nmol, 0.47 µg) and **2** (14.0 nmol, 1.24 µg) were added. The reaction mixture was shaken overnight at 25 °C to give conjugate **15**. MS (Accu-TOF) measurement gave 8599 Da as the main peak after deconvolution.

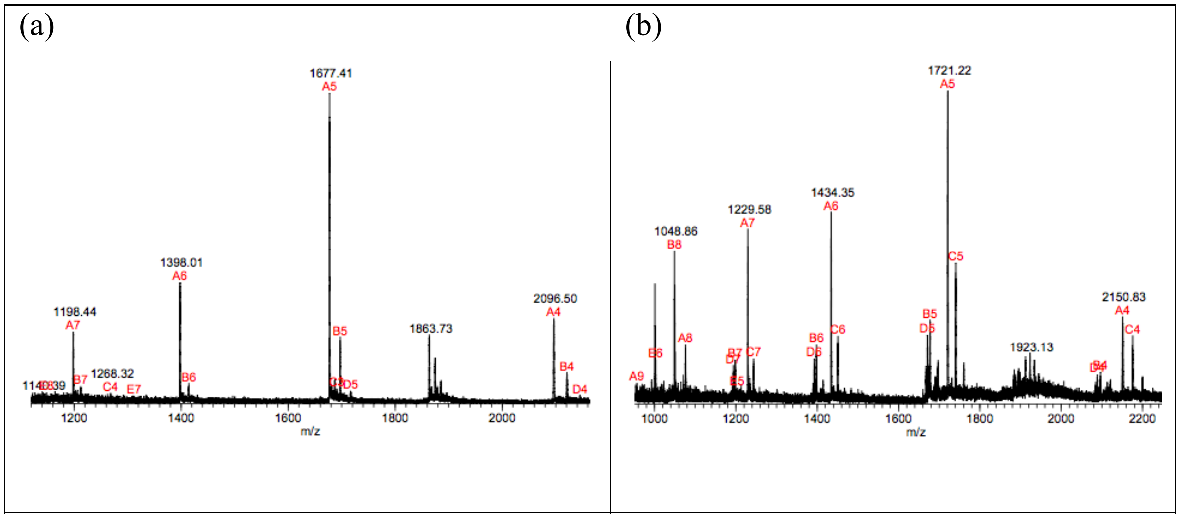

Figure 2. Oxidation and PEGylation of IL-8 following the one-pot protocol. (a) Native IL-8 (MW after deconvolution = 8382 Da). (b) IL-8 after oxidation, conjugation with **16**.

IL-8 labeling with PEG-cyclooctyne 16

A solution of IL-8 (5 µg, 0.60 nmol) in PBS buffer (10 µL) was diluted with NH_4OAc buffer pH 6.9 (10 µL). Subsequently, $NaIO_4$ (0.66 nmol, 0.14 µg) was added. The reaction was allowed to take place for 1h at room temperature, and 4-MeOPhSH was added (3.9 nmol, 0.55 µg, 0.5 nL). After stirring for 1 h at room temperature, p-anisidine (5.6 nmol, 0.69 µg) was added. After stirring for 15 min at room temperature, N-methylhydroxylamine hydrochloride (5.6 nmol, 0.47 µg) and **16**[12] (14.0 nmol, 28 µg) were added. The reaction mixture was shaken overnight at 25 °C to give conjugate **17**. The product was analysed by RP-HPLC. Retention time IL-8 11.54 min; retention time PEGylated IL-8 (**17**) 13.01 min.

[1] A. K. Parhi, W. Franck, *Org. Lett.* **2004**, 6, 3063-3065.

[2] L. Tian, G. Xu, L. Liu, *Synthesis* **2003**, 1329-1334.

[3] C. M. Dicken, P. DeShong, *J. Org. Chem.* **1982**, 47, 2047-2051.

[4] H. Song, C. J. Lim, S. Lee and S. Kim, *Chem. Commun.* **2006**, 2893-2895.

[5] X. Ning, J. Guo, M. A. Wolfert and G.-J. Boons, *Angew. Chem. Int. Ed.* **2008**, *47*, 2253-2255.

[6] N. J. Angelino, R. J. Bernacki, M. Sharma, O. Dodson-Simmons, W. Korytnyk, *Carbohydr. Res.* **1995**, *276*, 99-115.

[7] C. A. A. van Boeckel, T. Beetz, J. N. Vos, A. J. M. de Jong, S. F. van Aelst, R. H. van den Bosch, J. M. R. Mertens, F. A. van der Vlugt, *J. Carbohydr. Chem.* **1985**, *4*, 293-321.

[8] C. L. Jacobs, S. Goon, K. J. Yarema, S. Hinderlich, H. C. Hang, D. H. Chai, C. R. Bertozzi, *Biochemistry* **2001**, *40*, 12864-12874.

[9] V. Eschenfelder, R. Brossmer, *Carbohydr. Res.* **1980**, *78*, 190-194.

[10] Commercially available from Sigma-Aldrich.

[11] Commercially available from Genscript.

[12] M. F. Debets, S. S. van Berkel, S. Schoffelen, F. P. J. T. Rutjes, J. C. M. van Hest, F. L. van Delft, *Chem. Commun.* **2010**, *46*, 97-99.
